# FBRSL1 regulates the expression of chromatin regulators *BRPF1* and *KAT6A*

**DOI:** 10.1007/s00439-025-02760-y

**Published:** 2025-07-14

**Authors:** Gina Kastens, Hanna Berger-Santangelo, Sarah Gerstner, Roser Ufartes, Michaela Mischak, Annette Borchers, Silke Pauli

**Affiliations:** 1 Institute of Human Genetics, University Medical Center Göttingen, Heinrich-Düker-Weg 12, 37073 Göttingen, Germany; 2 Department of Biology, Molecular Embryology, Marburg University, Karl-von-Frisch Str. 8, 35043 Marburg, Germany; 3 Synaptic Systems GmbH, Rudolf-Wissell-Straβe 28a, 37079 Göttingen, Germany

## Abstract

*FBRSL1*-associated syndrome is a rare congenital malformation and intellectual disability syndrome caused by heterozygous truncating variants in *Fibrosin-Like 1* (*FBRSL1*). While *FBRSL1* is known to be involved in embryonic development, its precise molecular function remains poorly understood. Therefore, the aim of this study was to elucidate the molecular function of *FBRSL1*, which is thought to be essential for developmental processes, and to investigate the effect of patient-derived truncating *FBRSL1* variants. Using chromatin immunoprecipitation followed by sequencing (ChIP-Seq), we show that FBRSL1 regulates the expression of epigenetic regulators. We demonstrate that FBRSL1 associates with the transcription factor Yin Yang 1 (YY1) and binds upstream of *Bromodomain And PHD Finger containing 1* (*BRPF1*) and *Lysine Acetyltransferase 6 A* (*KAT6A*), two epigenetic regulators involved in embryonic development and linked to neurodevelopmental disorders. Furthermore, quantitative real-time PCR analysis revealed that truncating *FBRSL1* variants lead to downregulation of *BRPF1* and *KAT6A* in blood and fibroblasts derived from patients with the *FBRSL1*-associated syndrome. Consistently, loss of *Fbrsl1* function in *Xenopus laevis* embryos, which results in a range of developmental abnormalities, including craniofacial and brain malformations, also leads to defects in the *brpf1* and *kat6a* expression pattern. In summary, our findings support a function of *FBRSL1* in regulating key genes involved in global epigenetic processes and embryonic development. These results provide mechanistic insights how FBRSL1 dysfunction contributes to the pathogenesis of *FBRSL1*-associated syndrome.

**Supplementary Information:**

The online version contains supplementary material available at 10.1007/s00439-025-02760-y.

## Introduction

Monogenic developmental disorders (DDs) arise from pathogenic variants in single genes that are involved in fundamental processes during embryonic development. A significant proportion of genes associated with DD encode epigenetic modifiers, which regulate gene expression in a tightly coordinated manner through mechanisms such as DNA methylation, histone modification (e.g., acetylation and methylation) or ATP-dependent chromatin alteration (Ausió et al. 2003; Hota and Bruneau 2016; Mossink et al. 2021). Representative examples of rare monogenic DDs resulting from pathogenic variants in epigenetic regulators are Arboleda-Tham syndrome (ARTHS) (*KAT6A*; OMIM #616268) and intellectual developmental disorder with dysmorphic facies and ptosis (IDDDFP) (*BRPF1*; OMIM #617333).

Another example of a monogenic DD is the recently described *FBRSL1*-associated syndrome (Ufartes et al. 2020). The clinical phenotype comprises global developmental delay, respiratory distress and feeding difficulties in the neonatal period, no active speech, postnatal microcephaly, growth restriction, contractures and a variable degree of inner organ malformations like heart defects. Four patients have been described so far carrying heterozygous, truncating variants in *Fibrosin-Like 1* (*FBRSL1*) (Ufartes et al. 2020; Bukvic et al. 2024). *FBRSL1* is known to express alternatively spliced isoforms. In addition to a canonical long isoform consisting of 17 exons (NM_001142641.2), additional N-terminal isoforms have been described. Two short N-terminal isoforms (NM_001382741.1, NM_001382742.1) have been identified that contain an alternative exon 3, which introduces a premature stop codon (Ufartes et al. 2020). Notably, one patient variant is located within this alternative exon 3 and therefore affects only the N-terminal isoforms. This suggests that not only the canonical, but also the short N-terminal isoforms of FBRLS1 may play a critical role in embryonic development (Ufartes et al. 2020).

Knockdown studies in *Xenopus laevis* indicate that Fbrsl1 is required for the development of the brain, craniofacial structures and the heart (Ufartes et al. 2020; Berger et al. 2024). Immunofluorescence analysis in HEK293 cells and human fibroblasts showed that FBRSL1 is predominantly localized to the nucleus and depending on the isoform to mitotic spindles (Ufartes et al. 2020). These observations suggest that FBRSL1 might have different cellular functions depending on its isoform, leading to a multisystemic disorder when mutated.

The *Autism Susceptibility Candidate 2* (*AUTS2*) gene is a paralogue of *FBRSL1* (Sellers et al. 2020). Pathogenic variants in *AUTS2* are associated with a neurodevelopmental disorder called the *AUTS2* syndrome (Beunders et al. 2013). Interestingly, the phenotype associated with the *AUTS2* syndrome and the *FBRSL1*-associated syndrome overlaps to a considerable extent (Pauli et al. 2021). AUTS2 has been demonstrated to be multifunctional, notably playing a role in regulating the expression of (neuro)developmental genes (Oksenberg et al. 2014; Biel et al. 2022). In addition, interaction analysis showed that AUTS2 regulates gene expression through binding to non-canonical polycomb repressive complexes (PRCs) 1.3 and 1.5, which belong to the group of chromatin remodeling complexes (Gao et al. 2012; Fair et al. 2023). For FBRSL1, an association with PRC1.3 and PRC1.5 has also been described, but its nuclear function remains unclear (Gao et al. 2012).

In this study, we searched for genomic targets of FBRSL1 using chromatin immunoprecipitation followed by sequencing (ChIP-Seq) and investigated the effect of truncating *FBRSL1* variants on gene expression in blood and fibroblasts from patients with the *FBRSL1-*associated syndrome using quantitative real-time PCR (qPCR). In addition, we validated our data by performing in situ hybridizations in *Xenopus laevis* embryos.

## Methods

### Human cell culture and transfection

HEK293 and HeLa cells were cultured in Dulbecco’s Modified Eagle Medium (DMEM) (Thermo Fisher Scientific), supplemented with 10% fetal bovine serum (FBS) (PAN-Biotech), 1% penicillin/streptomycin (Thermo Fisher Scientific), and 1% MEM Non-Essential Amino Acids (NEAA) (Thermo Fisher Scientific) at 37 °C in a 5% CO_2_ incubator. HEK293 and HeLa cells were regularly tested for mycoplasma contamination using the MycoSPY^®^ PCR kit (Biontex).

### Co-immunoprecipitation

For co-immunoprecipitation (Co-IP) experiments, 1.6 × 10^6^ HEK293 cells were plated into T25 flasks. The next day, cells were transfected with either 4 µg of HA-FBRSL1-I1-pcDNA3.1 (NM_001142641.2) or HA-FBRSL1-I3.1-pcDNA3.1 (NM_001382741.1) (Ufartes et al. 2020) using jetPrime (Polypus) following the manufacturer’s instructions. Whole protein lysate was obtained 24 h after transfection using a modified RIPA buffer (1% NP-40, 0.25% sodium deoxycholate, 150 mM sodium chloride, 1 mM EDTA, 1X cOmplete™ protease inhibitor cocktail in ddH_2_O). Protein lysate was pre-cleared by incubating 400 µg protein diluted in PBS with 20 µL protein G magnetic beads (New England Biolabs) on a rotator for three hours at 4 °C. For Co-IP, pre-cleared protein lysate was incubated with either 0.2 µg of monoclonal anti-HA antibody (C29F4, Cell signaling), polyclonal anti-YY1 antibody (22156-1-AP, Proteintech), or anti-rabbit IgG (111-035-045, Jackson ImmunoResearch) in 800 µL IP buffer (20 mM HEPES, 100 mM potassium chloride, 5 mM EGTA, 5 mM magnesium chloride, 1% triton X-100, 5 mM PMFS, 1 mM dithiothreitol, 1X cOmplete™ protease inhibitor cocktail in ddH_2_O, pH 7.2) on a rotator for three hours at 4 °C. After adding 20 µL of the magnetic beads, samples were incubated for two hours at 4 °C with gentle rotation. Supernatant fractions were obtained before the samples were washed six times with wash buffer (50 mM TRIS, 5 mM magnesium chloride, 150 mM sodium chloride, 1% triton X-100 in ddH_2_O, pH 7.5). The immunocomplexes were boiled in 1X NuPAGE™ LDS sample buffer (Invitrogen) and 0,1 M dithiothreitol. Each experiment was carried out in two biological replicates.

### Western blot

Protein samples were separated in a 4–12% SurPAGE Bis–Tris Gel (Genscript) at 80 V for 10 min followed by 140 V for 60 min. Separated proteins were transferred to a 0.45 μm nitrocellulose membrane (Sigma-Aldrich) for 15 min at 150 mA followed by 60 min at 250 mA and 4 °C. The membrane was blocked with 2% Bovine Serum Albumin (BSA) in PBS with 0.1% Tween^®^ (PBST) 20 for one hour at room temperature. Incubation with primary antibody was performed overnight at 4 °C in 2% BSA/PBST. Incubation of secondary antibodies conjugated with horseradish peroxidase (HRP) was done for one hour at room temperature in 2.5% milk/TBST. Chemiluminescence signal was analyzed after adding the Immobilon^®^ Western Chemiluminescent HRP Substrate (Merck) in an AZURE c300 chemiluminescent western blot imager (Azure Biosystems). The following antibodies were used: monoclonal anti-HA (C29F4, Cell signaling, 1:2000), polyclonal anti-YY1 (22156-1-AP, Proteintech, 1:1000), and anti-rabbit IgG-HRP (A8275, Sigma, 1:10,000).

### Proximity-ligation assay

For proximity-ligation assay (PLA), 5 × 10^4^ HeLa cells were seeded onto 12 mm coverslips coated with 0.1% poly-L-lysine in a 24-well plate. The following day, HeLa cells were transfected with 0.25 µg of either HA-FBRSL1-I1-pcDNA3.1 (NM_001142641.1) or HA-FBRSL1-I3.1-pcDNA3.1 (NM_001382741.1) (Ufartes et al. 2020) along with 0.25 µg Flag-YY1-pcDNA3.1 (NM_003403.5) using jetPrime (Polypus). The Flag-YY1-pcDNA3.1 was a gift from Richard Young (Addgene plasmid #104396; http://n2t.net/addgene:104396; RRID: Addgene_104396) (Weintraub et al. 2017). The day after transfection, cells were washed with PBS and fixed with 4% paraformaldehyde for 10 min at room temperature. After washing three times with PBS, cells were incubated with PBST containing 0.5% triton X-100 for 10 min, followed by two incubation steps in PBST and 0.1% triton X-100 for 5 min. Blocking of non-specific antibody binding was performed by incubating the cells with 3% BSA in PBST and 0.1% triton X-100 for one hour at room temperature. Incubation of cells with primary antibodies diluted in 3% BSA/PBST was done in a wet chamber at 4 °C overnight. Following antibodies were used: monoclonal anti-HA (C29F4, Cell signaling, 1:8000) and monoclonal anti-Flag M2 (F1804, Sigma-Aldrich, 1:500). The next day, Duolink^®^ PLA was performed using the Duolink^®^ in situ anti-mouse (minus) and anti-rabbit (plus) PLA probes (Sigma-Aldrich) according to the manufacturer’s instructions. Cells were additionally stained with phalloidin-FITC (1:500) for 30 min at room temperature and mounted with Fluoroshield™ mounting medium (Sigma-Aldrich) with DAPI to visualize cell nuclei. Olympus FV1000 confocal laser scanning microscope (EVIDENT) and the FV10-ASW viewer software 4.2 (EVIDENT) were used for immunofluorescence analysis. Image editing was performed using ImageJ Fiji version 1.54f (National Institutes of Health) (Schindelin et al. 2012).

### ChIP-Seq

ChIP-Seq experiments were performed at Active Motif (https://www.activemotif.com/). Briefly, 5 × 10^6^ HEK293 cells were plated into T75 flasks the day before transfection. Cells were transfected with either 10 µg of HA-FBRSL1-I1-pcDNA3.1 (NM_001142641.2) (Ufartes et al. 2020) or empty HA-pcDNA3.1 vector using jetPrime (Polypus) following the manufacturer’s instructions. The next day, cells were fixed with 11% formaldehyde and harvested according to the ChIP Cell Fixation protocol provided by Active motif. Each ChIP-Seq experiment was performed in two biological replicates and frozen cell pellets were sent to Active Motif for further processing. Immunoprecipitation was performed using 2 µg of polyclonal anti-FBRSL1 antibody (HPA049880, Sigma) per 40 µg chromatin. Raw data processing and analysis were performed by Active motif. Human reference genome GRCh38 (hg38) was chosen for read alignment and peak calling was performed using MACS 2.1.0. Peaks were visualized using the Integrative Genomics Viewer (IGV) version 2.9.4 (Thorvaldsdóttir et al. 2013). The dataset of YY1 ChIP-Seq peaks in HEK293 cells was downloaded from the ENCODE database (https://www.encodeproject.org/) (ENCODE Project Consortium 2012; Luo et al. 2020; Hitz et al. 2023) with the accession number ENCSR859RAO. Known transcription factor motifs within FBRSL1 peaks were detected with *findMotifsGenome* of the HOMER package (http://homer.ucsd.edu/homer/). To identify FBRSL1 target genes enriched in Gene Ontology (GO) terms for molecular function, over-representation analysis (ORA) was performed using WebGestalt (Zhang et al. 2005; Elizarraras et al. 2024). FBRSL1 target genes detected in both replicates and filtered for peaks ± 5 kb from the transcription start site were analyzed using the Benjamini-Hochberg procedure with a false-discovery rate (FDR) set to 0.05.

### Patient fibroblasts

The patient’s fibroblast cell line was established from a skin biopsy of a 14-year-old patient harboring the heterozygous *FBRSL1* variant c.487C > T (p.Gln163*) (NM_001142641.2). Informed consent was obtained. The study has been exempted from ethical review by the Medical Ethical Review Committee of the UMG. The fibroblasts were cultured in DMEM (Thermo Fisher Scientific), supplemented with 10% FBS (PAN-Biotech), 1% penicillin/streptomycin (Thermo Fisher Scientific), and 1% NEAA (Thermo Fisher Scientific) at 37 °C in a 5% CO_2_ incubator. Early passages were treated with 1.5 µg/mL amphotericin B (Thermo Fisher Scientific). Cells were passaged or harvested when cell confluency reached approximately 90%.

### RNA isolation and reverse transcription

RNA isolation from cultured cells was performed using the NucleoSpin^®^ RNA mini kit (Macherey-Nagel) according to the manufacturer’s instructions. For the reverse transcription of RNA into complementary DNA (cDNA), the Tetro™ cDNA synthesis kit (Meridian Bioscience) was used. First, 1 µg RNA was incubated with random hexamer primers and nuclease-free water for 5 min at 70 °C. After adding the reverse transcription buffer, dNTPs and the reverse transcriptase, the reaction mixture was incubated for 30 min at 42 °C, followed by 5 min at 95 °C. The efficiency of cDNA synthesis was determined by PCR of the cDNA transcript of the housekeeper gene *TATA-Box Binding Protein* (*TBP*) and detection in a 2% agarose gel.

### Quantitative real-time PCR

The relative mRNA expression of FBRSL1 target genes was determined by qPCR using the QuantiTect SYBR^®^ Green PCR Kit (Qiagen). All cDNA samples were diluted 1:20 and measured in three technical replicates. The qPCR was performed using the 7900 HT Fast real-time PCR System (Applied Biosystems). The relative expression of target genes was calculated using the 2^−△△CT^ method. *Peptidylprolyl Isomerase B* (*PPIB*) served as internal control. The qPCR primers are listed in Supplementary Table 1.

### Statistical analysis

Statistical analyses were performed using the GraphPad Prism 8.0 software (https://www.graphpad.com). Bar charts were created using GraphPad Prism 8.0, displaying the mean and standard deviation. A Mann-Whitney U test was performed to compare the relative expression of FBRSL1 targets between patient blood or fibroblast samples and healthy controls determined in qPCR experiments. A value of *p* ≤ 0.05 was considered as statistically significant. One-way ANOVA followed by Tukey′s post hoc test was used to evaluate statistical significance of the *Xenopus laevis* experiments.

### *Xenopus* microinjection and analysis

All experiments involving *Xenopus laevis* embryos were conducted in accordance with the German Tierschutzgesetz and approved Regierungspräsidium Giessen (V 7/2022). Standard protocols were used to obtain and culture embryos, and developmental stages were defined according to Nieuwkoop and Faber (Nieuwkoop and Faber 1956). The investigators were blinded to the group allocation when they assessed the experimental outcome.

To knockdown Fbrsl1 in *Xenopus*, embryos were injected with a splice blocking morpholino (*fbrsl1* sp MO (Ufartes et al. 2020). As a control, a standard control morpholino (Co MO, 5′-CCTCTTACCTCAGTTACAATTTATA-3′) (Gene Tools, LLC) was used. Embryos were injected in the animal half of one blastomere at the two-cell stage with 10 ng of morpholino in combination with 80–100 pg *lacZ* mRNA or 80 pg *mGFP* mRNA for lineage tracing. *LacZ* (Smith and Harland 1991) and *mGFP* (Moriyoshi et al. 1996) mRNA were synthesized using the mMessage mMachine SP6 Transcription Kit (Invitrogen). For rescue experiments with human *BRPF1* and *KAT6A*, plasmids encoding GFP-BRPF1 (NM_001003694, Addgene plasmid #65382; http://n2t.net/addgene:65382; RRID: Addgene_65382, Gong et al. 2015) and Flag-HA hKAT6A (NM_006766.5, Weber et al. 2023) were co-injected together with 8 ng *fbrsl1* sp MO. For rescue experiments with the FBRSL1 isoforms, plasmids encoding human FBRSL1 isoform I1 (NM_001142641.2), isoform I3.1 (NM_001382741.1), and the variant isoform I3.1p.Q163* (Ufartes et al. 2020) were co-injected with the *fbrsl1* sp MO.

For spatial expression analysis, the embryos were fixed at stage 33 in MEMFA (3.7% formaldehyde, 0.1 M MOPS, 2 mM EGTA and 2 mM MgSO_4_) and analyzed by β-galactosidase staining and in situ hybridization (Smith and Harland 1991). To generate antisense and sense control in situ hybridization probes, *brpf1* (924 bp) and *kat6a* (977 bp) fragments were amplified by PCR from *Xenopus laevis* stage 33 cDNA using the primers listed in Supplementary Table 1. The PCR products were cloned into the pCS2 + vector, using the restriction enzymes BamHI and XhoI for *brpf1*, and ClaI and EcoRI for *kat6a*. Before analyzing *brpf1* and *kat6a* expression in *fbrsl1* morphants, the expression patterns of *brpf1* and *kat6a*, including sense controls, were determined for wild-type embryos at different stages of development (Supplementary Fig. S1).

## Results

### FBRSL1 binds to genomic regions, mainly upstream of coding genes

**Fig. 1:**
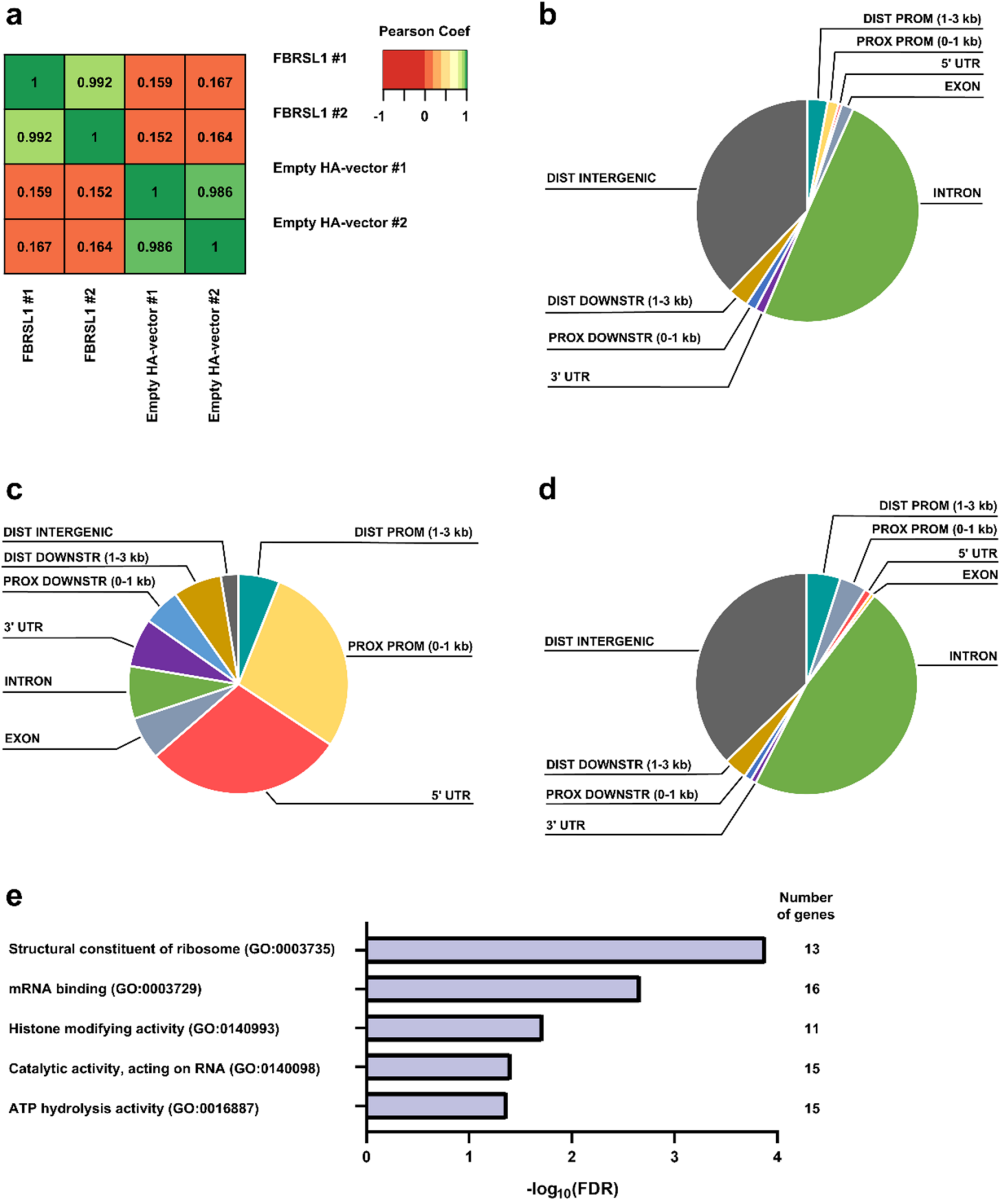
ChIP-Seq analysis of FBRSL1 targets after overexpression in HEK293 cells. **a** Heatmaps showing the Pearson correlation coefficient of the pairwise comparison of peak tag numbers. **b–d** Pie charts showing the localization of ChIP-Seq peaks relative to genomic annotations for (**b**) random control, (**c**) HA-FBRSL1-I1-pcDNA3.1, and (**d**) empty HA-pcDNA3.1 vector. The peak distribution shown represents one of the two biological replicates in each case. **e** Over-representation analysis (ORA) of FBRSL1 targets for Gene Ontology terms related to molecular function. Only FBRSL1 target genes with peaks in both FBRSL1 replicates located ± 5 kb from the transcription start site were considered. Benjamini-Hochberg procedure with a false-discovery rate set to 0.05 was performed using WebGestalt (https://www.webgestalt.org). *Coef* Coefficient, *DIST* Distal, *PROX* Proximal, *PROM* Promotor, *DOWNSTR* downstream, *UTR* untranslated region, *GO* Gene Ontology, *FDR* false-discovery rate

To identify genomic targets of FBRSL1, we performed ChIP-Seq analysis in HEK293 cells transfected with the long canonical HA-tagged FBRSL1 isoform (NM_001142641.2), further referred as I1. As control, HEK293 cells were transfected with an empty HA-vector. ChIP-Seq was initially attempted with an HA antibody but did not yield any results. Consequently, an FBRSL1-specific antibody detecting the overexpressed HA-tagged FBRSL1-I1 as well as the endogenous isoforms was used for immunoprecipitation. Using the Pearson Correlation Coefficient, it was shown that the peaks called in both biological replicates of overexpressed FBRSL1 exhibit a high degree of concordance indicating consistent FBRSL1 binding across samples (Fig. 1a). Similarly, a strong correlation was also observed for the peaks of the empty HA-vector. However, when comparing the FBRSL1 peaks with the peaks of the empty HA-vector, the Pearson Correlation Coefficient was below 0.2, suggesting a low concordance between FBRSL1 and control samples. Overall, these data suggest that we indeed identified FBRSL1-specific genomic binding sites.

In order to examine the localization of FBRSL1 targets across the genome, we compared the distribution pattern of FBRSL1 peaks and randomly distributed peaks (referred to as random control). We observed that FBRSL1 peaks were predominantly enriched in upstream regions of coding genes when compared to the peaks of the random control (Fig. 1b, c). In contrast, peaks of the vector control showed a distribution pattern similar to those of the random control indicating that these peaks result from unspecific binding (Fig. 1d). Only a slight enrichment of empty HA-vector peaks was detected in upstream regions of genes, partly overlapping with FBRSL1 peaks. Since HEK293 cells express FBRSL1 at a very low level, these few peaks upstream of genes could be explained by the binding of endogenous FBRSL1. In summary, these data revealed that the canonical FBRSL1 isoform I1 binds to the genome, mainly to upstream regions of coding genes. This observation suggests that FBRSL1 interacts directly or indirectly with regulatory sequences such as promoter regions to regulate gene expression.

We further analyzed the molecular function of FBRSL1 target genes by over-representation analysis (ORA). Target genes were selected based on the presence of FBRSL1 peaks within ± 5 kb of the transcription start site (TSS) that were consistently called in both replicates (Supplementary Table 2). ORA revealed that FBRSL1 predominantly binds to genes associated with RNA metabolism, ribosome assembly, ATP hydrolysis, and histone modifying and regulating proteins (Fig. 1e, Supplementary Table 3). Given the critical role of histone modifying processes during embryonic development, we focused our subsequent analysis on this functional category. Within this category, we identified *BRPF1* (*Bromodomain And PHD Finger Containing 1*) and *KAT6A* (*Lysine Acetyltransferase 6 A*), which encode chromatin regulating proteins that form the BRPF1-KAT6A complex (Viita and Côté 2022; Zu et al. 2022). As both *BPRF1* and *KAT6A* are known to be associated with monogenic DD, we concentrated our further experiments on these two FBRSL1 target genes.

### FBRSL1 associates with transcription factor YY1 and binds upstream of YY1 target genes

**Fig. 2:**
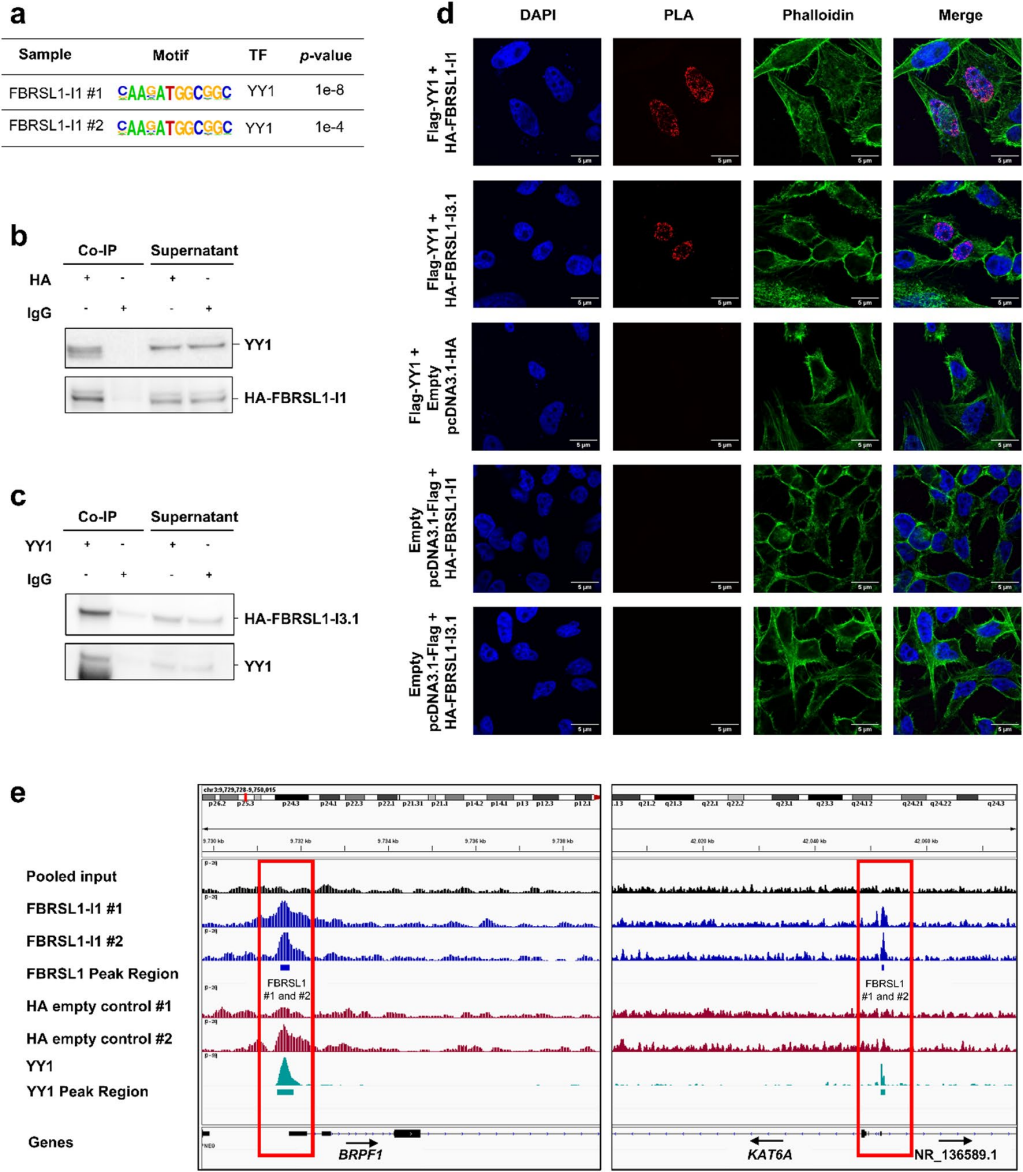
FBRSL1 binds to upstream regions of protein-coding genes that overlap with the YY1 binding motif and physically associates with YY1. **a** HOMER Motif analysis revealed an enrichment of YY1 transcription factor motif in FBRSL1-I1 binding regions. **b** Co-immunoprecipitation in HEK293 cells showed an association of endogenous YY1 with overexpressed HA-FBRSL1-I1. **c** Co-immunoprecipitation in HEK293 cells showed an association of endogenous YY1 with overexpressed HA-FBRSL1-I3.1. **d** Proximity-ligation assay of FBRSL1 isoforms I1 and I3.1, and YY1 in HeLa cells. **e** Visualization of FBRSL1-I1 and YY1 ChIP-Seq peaks upstream of *BRPF1* and *KAT6A*. Graphics were created using the Integrative Genome Browser (IGV) 2.9.4. *TF* transcription factor, *Co-IP* co-immunoprecipitation

To identify known motifs of transcription factors within FBRSL1 peak sequences, a motif analysis was performed. Among the most significantly enriched motifs in FBRSL1-I1 peak sequences in both replicates was the motif of the transcription factor YY1 (Yin Yang 1) (Fig. 2a, Supplementary Table 4). To confirm an association of the long canonical FBRSL1 isoform with YY1, we conducted a Co-IP with HEK293 cells overexpressing the HA-tagged FBRSL1-I1 using an anti-HA antibody. Subsequent western blot analysis of endogenous YY1 showed that FBRSL1-I1 is associated with this transcription factor (Fig. 2b). To evaluate whether the short N-terminal FBRSL1 isoform (NM_001382741.1), further referred to as I3.1, is also involved in transcriptional regulation via interaction with YY1, we additionally performed Co-IP for HA-tagged FBRSL1-I3.1 and YY1 (Fig. 2c). Indeed, our Co-IP results suggest that not only the long FBRSL1 isoform I1, but also the short N-terminal isoform I3.1, is associated with the transcription factor YY1. We further validated the interaction between YY1 and FBRSL1 isoforms I1 and I3.1 by performing a proximity ligation assay (PLA) using HeLa cells co-transfected with FBRSL1 isoforms and YY1 (Fig. 2d). As PLA signals were exclusively observed in the nucleus, we propose that the interaction is confined to the nuclear compartment. In addition, we examined the upstream regions of the FBRSL1 target genes *BRPF1* and *KAT6A*, and identified an overlap between FBRSL1-I1 and YY1 peaks (Fig. 2e).

### Truncating patient variants in *FBRSL1* lead to a downregulation of its target genes *BRPF1* and *KAT6A*

**Fig. 3:**
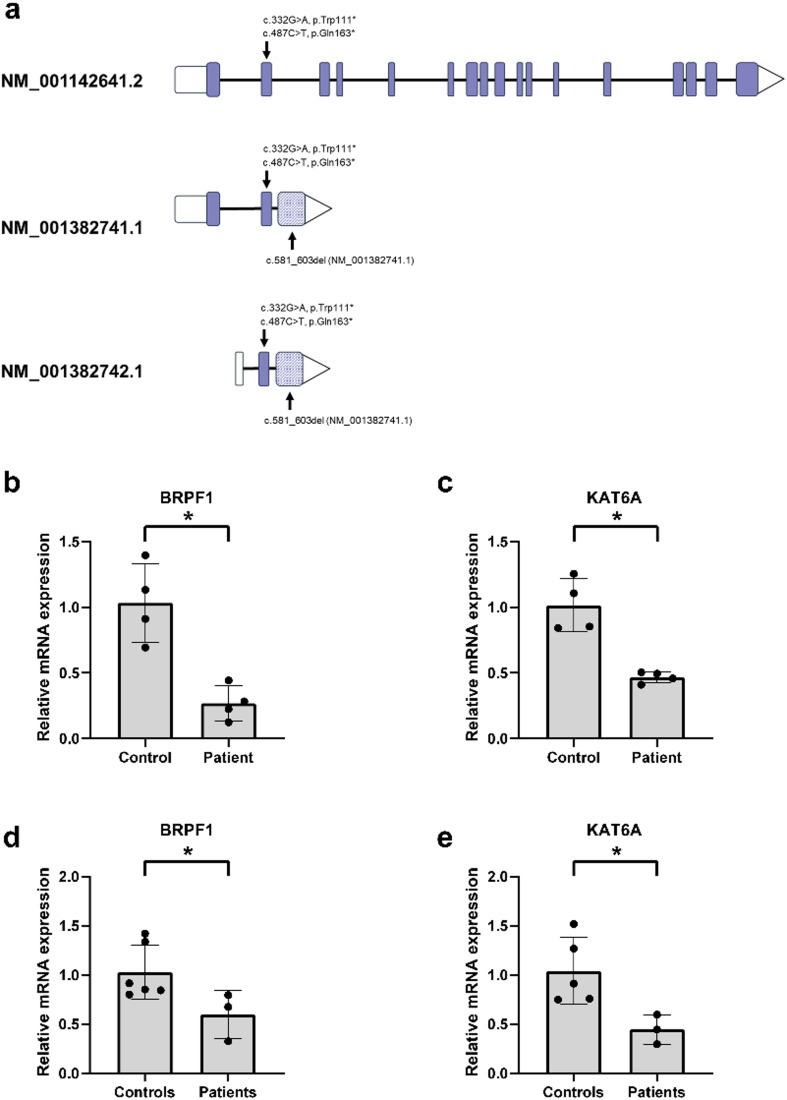
Truncating *FBRSL1* variants lead to a downregulation of its target genes in patient-derived fibroblasts. **a** Schematic presentation of the canonical long and the two short N-terminal FBRSL1 isoforms; truncating patient variants are indicated. Exon 3 (shaded) is an alternative exon ending with a stop codon, which is only present in the short N-terminal FBRSL1 isoforms. **b**, **c** qPCR analysis of FBRSL1 target genes in patient fibroblasts and healthy controls. Relative mRNA expression of (**b**) *BRPF1* and (**c**) *KAT6A* in fibroblasts from one patient harboring the heterozygous *FBRSL1* variant c.487C > T (p.Gln163*) compared to fibroblasts from a healthy control (*n* = 4). **d**, **e** qPCR analysis of FBRSL1 target genes (**d**) *BRPF1* and (**e**) *KAT6A* in blood samples from three patients harboring heterozygous *FBRSL1* variants c.487C > T (p.Gln163*), c.332G > A (p.Trp111*), and c.581_603del (*n* = 3) as well as the healthy controls (*n* = 5). The relative expression of target genes was determined using the 2^−△△CT^ method. Mann-Whitney U test was performed for statistical analysis. A value of *p* ≤ 0.05 was considered as statistically significant (*)

To examine the pathogenic effect of truncating variants in *FBRSL1* on the expression of its target genes, we determined the relative expression of *BRPF1* and *KAT6A* by qPCR analysis using patient-derived fibroblasts and blood. Fibroblasts were only available from one patient harboring the stop variant c.487C > T (p.Gln163*) (NM_001142641.2), while blood samples were available from three patients carrying the N-terminal located variants c.332G > A (p.Trp111*), c.487C > T (p.Gln163*) (NM_001142641.2), and c.581_603del (NM_001382741.1) (Fig. 3a). The qPCR analysis revealed a significant downregulation of the FBRSL1 targets *BRPF1* and *KAT6A* in both fibroblasts and blood from patients affected by *FBRSL1*-associated syndrome, compared to healthy age-matched controls (Fig. 3b-e).

### Clinical phenotype of patients with the *FBRSL1*-associated syndrome overlaps with that of patients with *BRPF1* or *KAT6A*-related syndromes 

Our qPCR results demonstrate that truncating variants in *FBRSL1* lead to a downregulation of *BRPF1* and *KAT6A*. These two chromatin regulators are associated with monogenic DDs. Pathogenic variants in *BRPF1* lead to the autosomal-dominant inherited IDDDFP, while pathogenic variants in *KAT6A* cause the autosomal-dominant inherited ARTHS (Arboleda et al. 2015; Mattioli et al. 2017; Yan et al. 2017). IDDDFP and ARTHS are predominantly caused by heterozygous, protein-truncating variants that result in a dysfunction of BRPF1 or KAT6A, respectively (Zu et al. 2022). A comparison of the clinical features of *FBRSL1*-associated syndrome with those of *BRPF1* and *KAT6A*-related syndromes reveals an overlap, especially of neurodevelopmental aspects (Table 1). Common features of neurodevelopmental disorders, such as intellectual disability (ID), global developmental delay, and microcephaly, but also more unique features are reported for all three syndromes. These include delayed speech development or absent speech, which is observed in all four patients with a pathogenic *FBRSL1* variant and is also a common feature in ARTHS (Ufartes et al. 2020; St John et al. 2022; Bukvic et al. 2024). Taken together, patients affected by *FBRSL1*-associated syndrome share features with IDDDFP and ARTHS patients. This observation might be explained by the downregulation of *BRPF1* and *KAT6A* in patients with truncating *FBRSL1* variants.

**Table 1:** Comparison of the clinical findings of the *FBRSL1*-associated syndrome with the phenotype of *KAT6A* and *BRPF1*-related syndromes

Clinical description (HPO term)	*FBRSL1*-associated syndrome	Arboleda-Tham syndrome (OMIM # 616268)	Intellectual developmental disorder with dysmorphic facies and ptosis (OMIM # 617333)
	(Ufartes et al. 2020; Pauli et al. 2021; Bukvic et al. 2024)	(Kennedy et al. 2019; St John et al. 2022; Vos et al. 2023)	(Mattioli et al. 2017; Yan et al. 2017, 2020; Souza et al. 2022; Colson et al. 2024)
Intellectual disability (HP:0001249) and/or global development delay (HP:0001263)	4/4	Common	Common
Delayed speech and language development (HP:0000750)	4/4	Common	Common
Autistic behavior (HP:0000729) and/or motor stereotypy (HP:0000733)	4/4	Common	Rare
Microcephaly (HP:0000252)	4/4	Common	Common (mild)
Postnatal growth retardation (HP:0008897)	4/4	Common	Common
Short stature (HP:0004322)	4/4	Case Reports (Arboleda et al. 2015; Urreizti et al. 2020; Wang et al. 2022)	Common
Camptodactyly/contractures (HP:0001371)	4/4	Rare	Common
Scoliosis and/or kyphosis	4/4	Not reported or extremely rare	Rare
Abnormal brain morphology (HP:0012443)	yes/no/NA/NA	Rare	Common
Seizure (HP:0001250)	1/4	Rare	Common
Motor/movement disorder (HP:0100022)	4/4	Common	Common
Feeding difficulties (HP:0011968)	4/4	Common	Common
Respiratory failure (HP:0002878)	3/4	Rare	Not reported or extremely rare
Heart defect (HP:0001627)	2/4	Common	Rare
Atrial/Ventricular septal defect (HP:0001631/HP:0001629)	2/4	Common	Rare
Cleft palate (HP:0000175)	2/4	Rare	Not reported or extremely rare
Asplenia (HP:0001746)	1/4	Not reported or extremely rare	Not reported or extremely rare
Abnormality of the anus (HP:0004378)	1/4	Not reported or extremely rare	Not reported or extremely rare
Hearing impairment (HP:0000365)	2/4	Rare	Not reported or extremely rare
Abnormality of the skin (HP:0000951): skin creases	2/4	Not reported or extremely rare	Not reported or extremely rare

Conditions occurring in 25% or more of cases are considered as common. Conditions are classified as rare when occurring in less than 25% of cases and as extremely rare when occurring in less than 1% of cases. *HPO* Human Phenotype Ontology, *NA* not assessed

### Fbrsl1 affects BRPF1 and KAT6A expression during *Xenopus* craniofacial development

Our data suggest that the *FBRSL1*-associated syndrome may cause a downregulation of *BRPF1* and *KAT6A* expression. As these target genes regulate epigenetic processes on a global scale during embryonic development, it is likely that the developmental defects observed in patients affected by *FBRSL1*-associated syndrome are in part caused by a dysregulation of their expression.

**Fig. 4:**
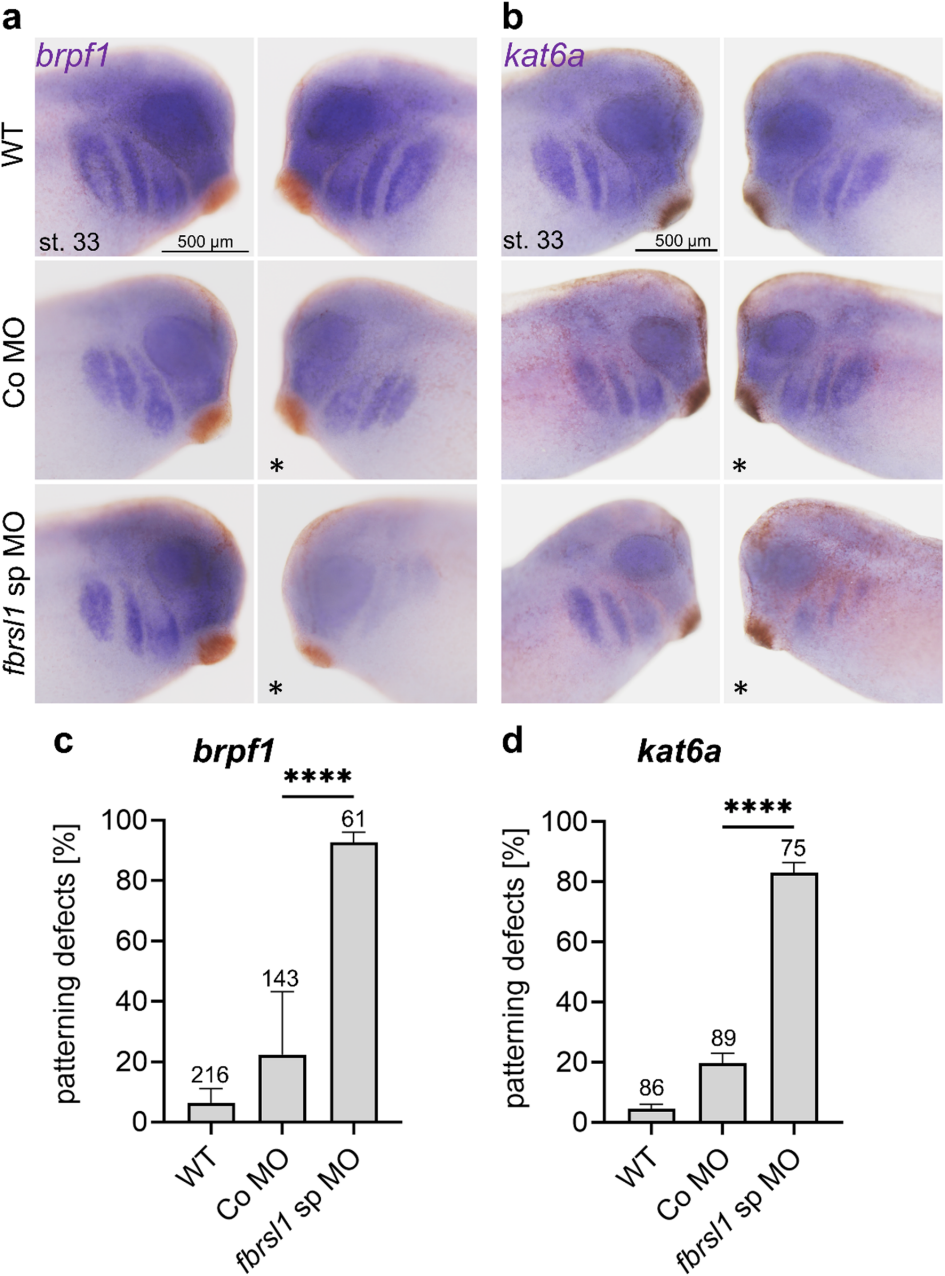
*Fbrsl1* depletion leads to defects in the *brpf1* and *kat6a* expression pattern in *Xenopus laevis* embryos. *Xenopus* embryos were injected with 10 ng splice blocking *fbrsl1* morpholino (*fbrsl1* sp MO) or control morpholino (Co MO) together with 100 pg *lacZ* or 80 pg *gfp* mRNA as a lineage tracer in one blastomere at the two-cell stage. At tadpole stages (stage 33), the *brpf1* or *kat6a* expression was analyzed by in situ hybridization. The injected side is indicated by a star. *WT* wild-type embryos. **a**,** c** Embryos analyzed for *brpf1* expression. **b**,** d** Embryos analyzed for *kat6a* expression. **c**, **d** Graphs summarize *brpf1* and *kat6a* patterning defects of four to six independent experiments. The bar charts show the mean value along with the standard error of the mean value. The number of embryos evaluated is indicated in all diagrams. One-way ANOVA followed by Tukey’s post hoc test was used to evaluate statistical significance (****p ≤ 0.0001). *WT* wild-type, *st* stage

To test this hypothesis, we turned to the *Xenopus laevis* system and first determined the *brpf1* and *kat6a* expression pattern. Both genes show expression in the premigratory and migratory neural crest cells, the brain as well as the eye from neural to tadpole stages (Supplementary Fig. S1). To test whether loss of Fbrsl1 function affects *brpf1* and *kat6a* expression, embryos were injected with either splice blocking *fbrsl1* morpholino oligonucleotides (*fbrsl1* sp MO) or control morpholino oligonucleotides (Co MO) in combination with a lineage tracer in one blastomere at the two-cell stage. Embryos were fixed at tadpole stages and the expression pattern of *brpf1* and *kat6a* was analyzed by in situ hybridization. Indeed, the embryos showed significant defects in the *brpf1* and *kat6*a expression pattern on the injected side in comparison to the uninjected side of tadpole stage embryos (Fig. 4a-d).

**Fig. 5:**
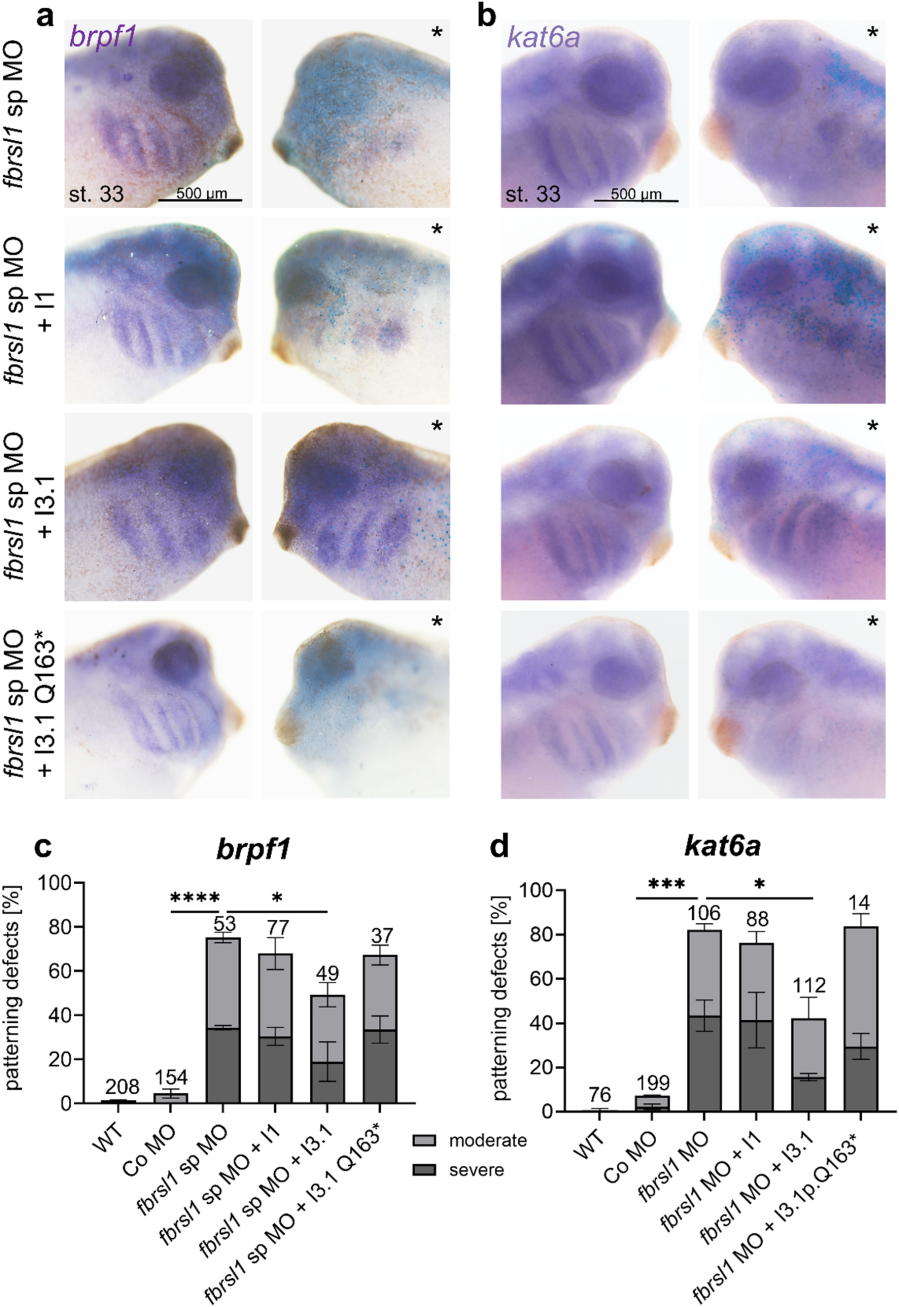
Defects of *brpf1* and *kat6a* expression patterns in *Xenopus laevis* morphant embryos can be rescued by co-injection of the short N-terminal *fbrsl1* isoform I3.1. At the two-cell stage, *Xenopus* embryos were injected in one blastomere with 10 ng of either a splice blocking *fbrsl1* morpholino (*fbrsl1* sp MO) or a control morpholino (Co MO, see Fig. 4), along with 100 pg of *lacZ* mRNA as a lineage tracer. For rescue experiments, human *FBRSL1* constructs I1 (NM_001142641.2) and I3.1 (NM_001382741.1) were co-injected. At tadpole stage (stage 33), *brpf1* or *kat6a* expression was examined using in situ hybridization. The injected side is marked with a star. The short N-terminal isoform I3.1 significantly restores the patterning defects of *brpf1* and *kat6a*, whereas the long isoform I1 and the short isoform, carrying one of the patient mutations (I3.1p.Q163*) did not rescue. **a**,** c** Embryos analyzed for *brpf1* expression. **b**,** d** Embryos analyzed for *kat6a* expression. **c**,** d** Graphs summarize *brpf1* and *kat6a* patterning defects. The bar charts show the mean value along with the standard error of the mean value. The number of embryos evaluated is indicated in all diagrams. One-way ANOVA followed by Tukey’s post hoc test was used to evaluate statistical significance (*p ≤ 0.05, ***p ≤ 0.001, ****p ≤ 0.0001). *WT* wild-type, *st* stage

To analyze the functional relevance of the different FBRSL1 isoforms in respect to the *brpf1* and *kat6a* expression pattern, we performed rescue experiments in *Xenopus laevis* embryos. The *fbrsl1* sp MO was co-injected with the human wild-type *FBRSL1* isoforms I1 and I3.1, as well as the patient-derived truncating variant I3.1p.Q163*. Subsequently, in situ hybridization was performed to assess the ability of the different isoforms to rescue the patterning defects of *brpf1* and *kat6a* caused by Fbrsl1 depletion (Fig. 5a-d). Co-injection of the N-terminal human *FBRSL1* isoform I3.1 significantly rescued the patterning defects of *kat6a* and *brpf1*, while neither the human canonical long isoform nor the truncating patient variant (I3.1p.Q163*) were able to rescue; the latter confirming the pathogenic effect of the variant.

**Fig. 6:**
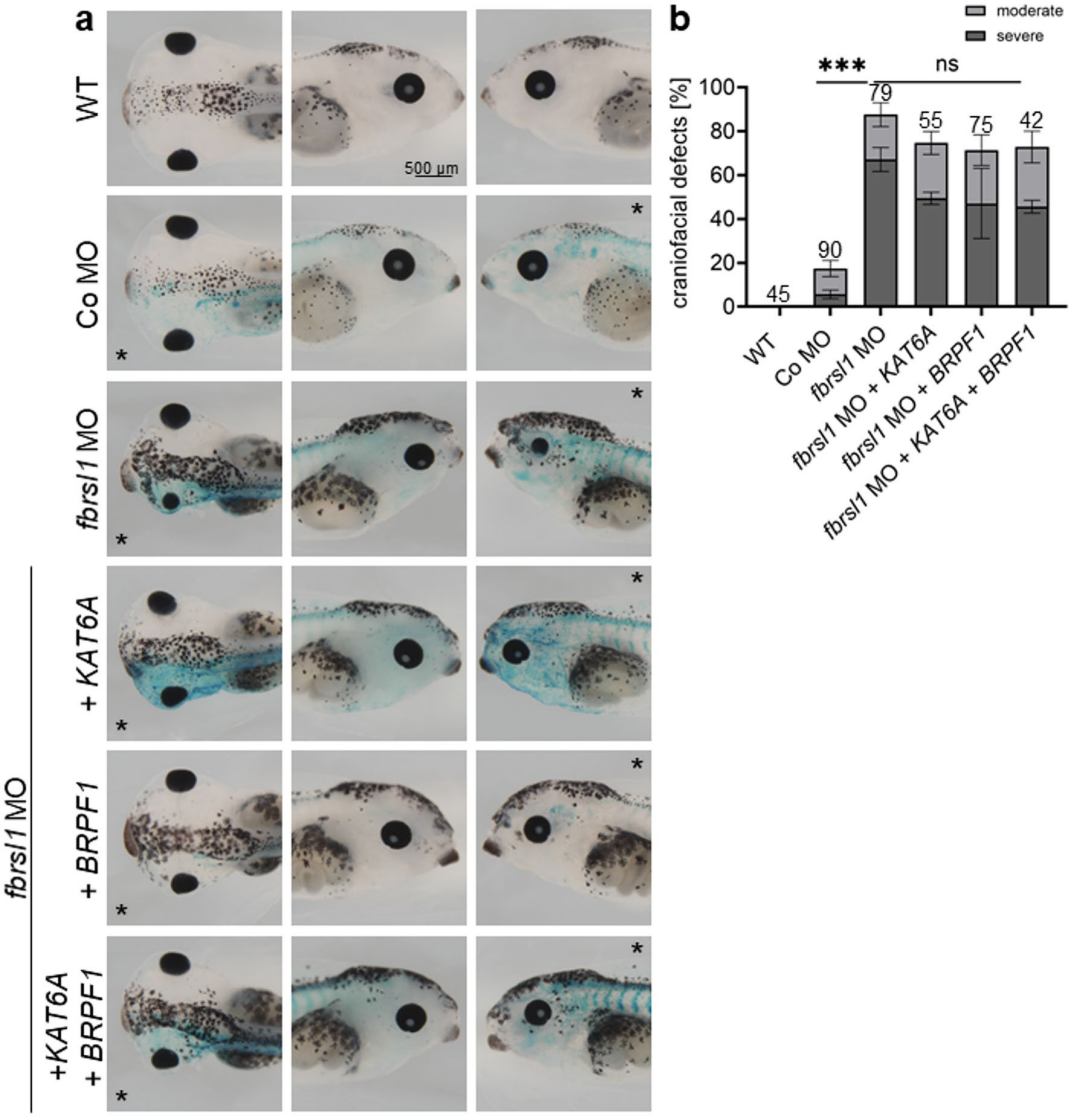
Co-injection of human *BRPF1* and *KAT6A* in *Xenopus laevis* embryos is not sufficient to rescue the craniofacial defects caused by *fbrsl1* depletion. *Xenopus* embryos were injected with the *fbrsl1* sp MO or Co MO in combination with plasmids containing human *BRPF1* and *KAT6A* in one blastomere at the two-cell stage as indicated. For lineage tracing, 80 pg *lacZ* mRNA was co-injected. The star marks the injected side. Embryos were phenotypically analyzed for craniofacial defects at stage 43. **a**,** b** Embryos injected with the *fbrsl1* MO show a strong reduction of craniofacial tissue on the injected side. Co-injection of embryos with human BRPF1 and KAT6A, alone or in combination, leads to a mild rescue of craniofacial defects. **b** Graph summarizes craniofacial defects of three independent experiments. The bar charts show the mean value along with the standard error of the mean value. The number of embryos evaluated is indicated in all diagrams. One-way ANOVA followed by Tukey’s post hoc test was used to evaluate statistical significance (***p ≤ 0.001). *WT* wild-type, *ns* not significant

Next, we tested whether BRPF1 and KAT6A are sufficient to rescue the *fbrsl1* craniofacial morphant phenotype. For this purpose, we co-injected the *fbrsl1* sp MO together with human *BRPF1* and *KAT6A* in one blastomere at the two-cell stage (Fig. 6a-b). Although the results were not statistically significant, we were able to see a mild improvement of craniofacial malformations at tadpole stages after co-injection of BRPF1 and KAT6A, either alone or in combination. Taken together, our analyses in the *Xenopus* model system support a role for FBRSL1 in regulating *BRPF1* and *KAT6A* expression, but also indicate broader functions, as supported by our ChIP-Seq data showing FBRSL1-associated regulation of genes across diverse pathways and cellular processes.

## Discussion

**Fig. 7:**
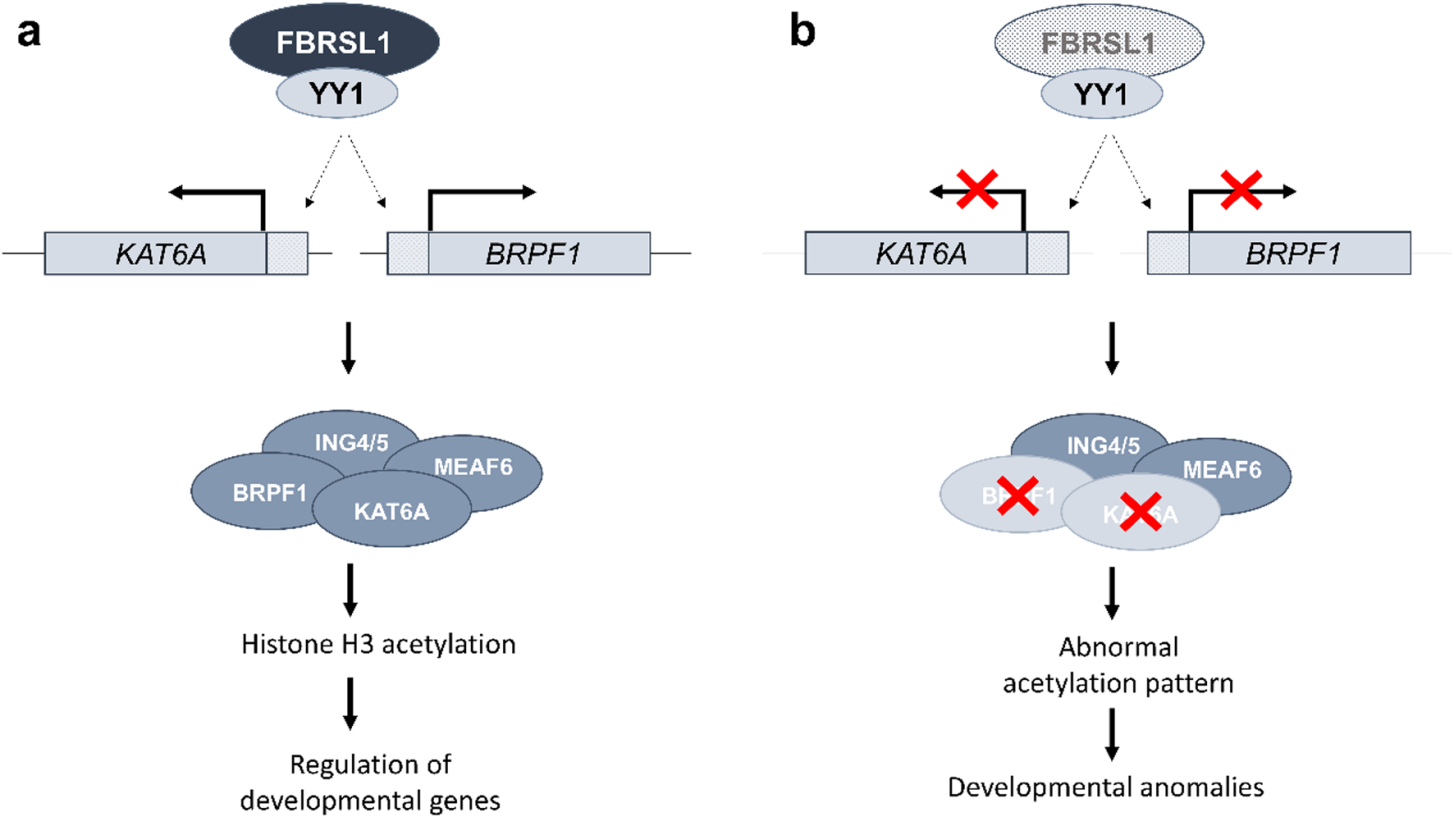
Hypothetical model of FBRSL1-mediated regulation of chromatin regulators *BRPF1* and *KAT6A* and their dysregulation in patients with truncating *FBRSL1* variants. **a** FBRSL1 regulates the expression of *BRPF1* and *KAT6A*, likely through its association with YY1. Along with ING4/5 and MEAF6, BRPF1 and KAT6A build the chromatin modifying BRPF1-KAT6A complex that controls the expression of developmental genes via histone H3 acetylation. **b** Truncating variants in *FBRSL1* (shaded) lead to a downregulation of *BRPF1* and *KAT6A* and therefore insufficient BRPF1-KAT6A complex activity. This likely causes an abnormal acetylation pattern and subsequently developmental abnormalities

The *FBRSL1*-associated syndrome is a rare monogenic DD characterized by global developmental delay, multiple dysmorphic features, and congenital organ malformations. Knockdown studies in *Xenopus laevis* have recently shown that Fbrsl1 is critical for various developmental processes, leading to craniofacial defects as well as heart anomalies when depleted (Ufartes et al. 2020; Berger et al. 2024). In this study, we examined the transcriptional function of *FBRSL1* to gain more insight into the pathomechanism of truncating variants in patients with *FBRSL1*-associated syndrome.

As previously reported by our group, *FBRSL1*-associated syndrome shares noticeable clinical overlap with *AUTS2* syndrome, another neurodevelopmental disorder associated with developmental delay, autistic features, epilepsy, microcephaly, and additional features like short stature, and craniofacial dysmorphisms (Beunders et al. 2013; Hori et al. 2021; Pauli et al. 2021). *AUTS2* syndrome is caused by pathogenic variants in *AUTS2*, a paralogous gene of *FBRSL1* (Sellers et al. 2020). AUTS2 is a multifunctional protein that has been shown to exert functions both in the nucleus and the cytoplasm (Biel et al. 2022). It regulates neuronal differentiation via complex transcriptional networks, modulates epigenetic landscapes through interaction with the PRC, and influences synaptogenesis and cytoskeletal dynamics by promoting lamellipodia and inhibiting filopodia formation (Gao et al. 2014; Hori et al. 2014; Monderer-Rothkoff et al. 2021). Furthermore, it is implicated in various aspects of RNA metabolism, including transcription, splicing, and stability, highlighting its broad functional spectrum (Castanza et al. 2021). Different AUTS2 isoforms are involved in the diverse functions to varying degrees, depending on their expression pattern and interaction network. In addition to a full-length isoform containing 19 exons, a short C-terminal isoform generated from an alternative transcription start site within exon 9 is described (Beunders et al. 2013). The short C-terminal isoform is exclusively expressed in the nucleus, while the full-length form is localized to the nucleus and cytoplasm (Hori et al. 2014). Zebrafish knockdown and rescue studies demonstrated that both the human short C-terminal isoform as well as the full-length AUTS2 are able to rescue the developmental defects, leading to the suggestion that both isoforms are necessary for neural development (Beunders et al. 2013; Oksenberg and Ahituv 2013).

Although the function of FBRSL1 is largely unknown, the fact that FBRSL1 and AUTS2 are paralogs and cause similar clinical phenotypes when mutated suggests that they are involved in similar cellular processes. For example the association with the nuclear PRC1.3 and PRC1.5 is described for both proteins (Gao et al. 2012). While the exact function of FBRSL1-PRC remains unexplored, AUTS2 has been shown to activate gene expression through its interaction with PRC1 (Gao et al. 2014; Liu et al. 2021). ChIP-Seq analyses have further revealed that AUTS2 is a transcriptional regulator of genes implicated in neurodevelopment (Gao et al. 2014; Oksenberg et al. 2014). It is therefore likely that FBRSL1 is also involved in the regulation of gene expression during developmental processes.

Our ChIP-Seq analysis in HEK293 cells showed that the long canonical FBRSL1 isoform I1 binds predominantly in upstream regions of protein-coding genes, indicating that FBRSL1 regulates gene expression by interaction with regulatory sites such as promotors. This binding might be indirect through the interaction with further transcription factors and complexes. In this study, we showed that FBRSL1 peaks overlap with the genomic binding motif of the transcription factor YY1, which is a key regulator of gene expression during embryonic development. Previous studies have shown that YY1 also interacts with components of the PRC1 (Kalenik et al. 1997; García et al. 1999; Basu et al. 2014). Therefore, it can be assumed that FBRSL1 regulates gene expression via its interaction with the YY1-PRC complex. Our protein-protein interaction analyses demonstrated that both the long canonical FBRSL1 isoform I1 and the N-terminal isoform I3.1 associates with YY1, implying that each may contribute to transcriptional regulation. Notably, ChIP-seq experiments of AUTS2 in mouse embryonic forebrains also showed an overlap of AUTS2 peaks and the YY1 recognition motif, providing additional support for our hypothesis that both paralogs are involved in shared cellular processes (Oksenberg et al. 2014).

By downstream enrichment analysis of FBRSL1 targets, we showed that FBRSL1-I1 binds to upstream regions of chromatin regulating genes, including *BRPF1* and *KAT6A*. Both target genes contain upstream YY1 binding motifs overlapping with FBRSL1 peaks, further supporting a coordinated regulatory mechanism of FBRSL1 and YY1 (Fig. 7).

To analyze how truncating *FBRSL1* variants affect the expression of its target genes, we performed qPCR analyses comparing *BRPF1* and *KAT6A* expression in fibroblasts and blood samples from patients and healthy controls. Indeed, *BRPF1* and *KAT6A* were significantly downregulated in patients with the *FBRSL1*-associated syndrome. BRPF1, a chromatin reader, and KAT6A, an acetyltransferase form together the BRPF1-KAT6A complex which regulates epigenetic processes during embryonic development (Viita and Côté 2022; Zu et al. 2022). It is therefore conceivable that a dysregulation of these genes leads to a global epigenetic dysregulation during embryonic processes resulting in various developmental defects (Fig. 7).

To validate the regulatory link between *FBRSL1* and *BPRF1* as well as *KAT6A*, we performed in situ hybridizations of *BRPF1* and *KAT6A* in *Xenopus laevis* embryos after knockdown of *fbrsl1* and observed significant defects in their expression patterns. Our rescue studies showed that co-injection of the short N-terminal FBRSL1 isoform I3.1 significantly rescues the patterning defects of *BRPF1* and *KAT6A*, while the long FBRSL1 isoform I1 and the mutant isoform I3.1p.Q163* did not rescue.

For both target genes, *BPRF1* and *KAT6A*, knockdown studies in zebrafish showed an involvement in craniofacial development (Miller et al. 2004; Crump et al. 2006; Laue et al. 2008). To assess whether the downstream targets *BRPF1* and *KAT6A* can rescue craniofacial defects due to fbrsl1 depletion in *Xenopus laevis*, we performed further rescue studies by co-injecting human *BRPF1* and *KAT6A* in *fbrsl1*-depleted embryos. Although not statistically significant, a mild rescue could be achieved. These results indicate that the developmental defects resulting from loss of *fbrsl1* are not solely due to the dysregulation of these two target genes, but are more likely caused by the dysregulation of a broader network of genes involved in craniofacial development. Consistent with this, our ChIP-Seq data showed that FBRSL1 not only regulates chromatin regulating genes, but also genes associated with other GO terms such as “structural constituent of ribosome” or “mRNA binding”. In addition to this, it is assumed that FBRSL1, similar to its paralog AUTS2, is a multifunctional protein with additional functions beyond transcriptional regulation that may be critical for embryonic development (Ufartes et al. 2020).

Clinically, the *FBRSL1*-associated syndrome shares several phenotypic features with *BRPF1*- and *KAT6A*-related syndromes, particularly neurodevelopmental defects. However, there are also distinct symptoms observed exclusively in patients with the *FBRSL1*-associated syndrome that are not present in patients with IDDDFP or ARTHS. This clinical observation is well in line with our experimental findings, supporting the hypothesis that the syndromic phenotype caused by truncating *FBRSL1* variants is not limited to the dysregulation of the expression of *BRPF1* and *KAT6A*, but rather involves a more complex interplay of disrupted cellular pathways and gene networks.

In conclusion, our in vitro studies revealed that FBRSL1 regulates the expression of important chromatin regulating genes such as *BRPF1* and *KAT6A*. We showed that pathogenic variants in *FBRSL1* lead to a downregulation of *BRPF1* and *KAT6A* in patients with the *FBRSL1-*associated syndrome. Future studies focusing on the specific roles of different FBRSL1 isoforms in distinct cellular processes will be key to further unraveling the complex clinical phenotype observed in patients with the *FBRSL1*-associated syndrome.

## Supplementary Information

Below is the link to the electronic supplementary material.

Supplementary material 1 (PDF 291.7 kb)

Supplementary material 2 (PDF 173.6 kb)

Supplementary material 3 (XLSX 12.8 kb)

Supplementary material 4 (XLSX 19661.4 kb)

Supplementary material 3 (PDF 408.7 kb)

## Data Availability

The datasets generated during the current study and/or analyzed are available from the authors upon reasonable request.
