# Supplementary material for: FBRSL1 regulates the expression of chromatin regulators BRPF1 and KAT6A
Source: Hum Genet. 2025 Jul 14;144(8):809–26. doi: 10.1007/s00439-025-02760-y (PMC12449339; doi:10.1007/s00439-025-02760-y)
Supplement: Supplementary file 1 — Supplementary material 1 (PDF 291.7 kb) [file 439_2025_2760_MOESM1_ESM.pdf]

FBRSL1 regulates the expression of chromatin regulators *KAT6A* and *BRPF1*

Human Genetics

Gina Kastens[1], Hanna Berger-Santangelo[2], Sarah Gerstner[2], Roser Ufartes[1,3], Annette Borchers[2]*, Silke Pauli[1]*

[1]Institute of Human Genetics, University Medical Center Göttingen, Heinrich-Düker-Weg 12, 37073 Göttingen, Germany

[2]Department of Biology, Molecular Embryology, Philipps-University Marburg, Karl-von-Frisch Str. 8, 35043 Marburg, Germany

[3]Synaptic Systems GmbH, Rudolf-Wissell-Straβe 28a, 37079 Göttingen, Germany.

*Corresponding authors

Silke Pauli

E-mail address: silke.pauli@med.uni-goettingen.de

Annette Borchers

E-mail address: borchers@uni-marburg.de, ORCID-ID: 0000-0002-2524-5384

Suppl. Table 1 List of primers used for qPCR and *in situ* hybridization experiments.

Species	Target	Application	Primer		Sequence	
H. sapiens	PPIB	qPCR	PPIB-F	5'	GCACAGGAGGAAAGAGCATC	3'
			PPIB-R	5'	TGAAGAACTGGGAGCCGTTG	3'
H. sapiens	BRPF1	qPCR	BRPF1-F	5'	ATGTTTCTGCGAGCACCACT	3'
			BRPF1-R	5'	CTCTGTCTTCCGACGCTCATT	3'
H. sapiens	KAT6A	qPCR	KAT6A-F	5'	CCCACAGACAATCAGGATGG	3'
			KAT6A-R	5'	CTCAATGACAGAGGGACAGC	3'
X. laevis	brpf1	*in situ* hybridization	Fw_xKat6a	5'	TAAGCAATCGATATGGTAAAACTGGCAAACCCCTTG	3'
			Rev_xKat6A	5'	TGCTTAGAATTCCGAATCTGTGCAGCCTTTTTGTG	3'
X. laevis	kat6a	*in situ* hybridization	Fw_xBrpf1	5'	TAGGATCCGAACCCAAAAACGGCAGGAC	3'
			Rev_xBrpf1	5'	TACTCGAGCCCTGCACTTTGTTGCGATG	3'
