# Supplementary material for: FBRSL1 regulates the expression of chromatin regulators BRPF1 and KAT6A
Source: Hum Genet. 2025 Jul 14;144(8):809–26. doi: 10.1007/s00439-025-02760-y (PMC12449339; doi:10.1007/s00439-025-02760-y)
Supplement: Supplementary file 2 — Supplementary material 2 (PDF 173.6 kb) [file 439_2025_2760_MOESM2_ESM.pdf]

FBRSL1 regulates the expression of chromatin regulators *KAT6A* and *BRPF1*

Human Genetics

Gina Kastens[1], Hanna Berger-Santangelo[2], Sarah Gerstner[2], Roser Ufartes[1,3], Annette Borchers[2]*, Silke Pauli[1]*

[1]Institute of Human Genetics, University Medical Center Göttingen, Heinrich-Düker-Weg 12, 37073 Göttingen, Germany

[2]Department of Biology, Molecular Embryology, Philipps-University Marburg, Karl-von-Frisch Str. 8, 35043 Marburg, Germany

[3]Synaptic Systems GmbH, Rudolf-Wissell-Straβe 28a, 37079 Göttingen, Germany.

*Corresponding authors

Silke Pauli

E-mail address: silke.pauli@med.uni-goettingen.de

Annette Borchers

E-mail address: borchers@uni-marburg.de, ORCID-ID: 0000-0002-2524-5384

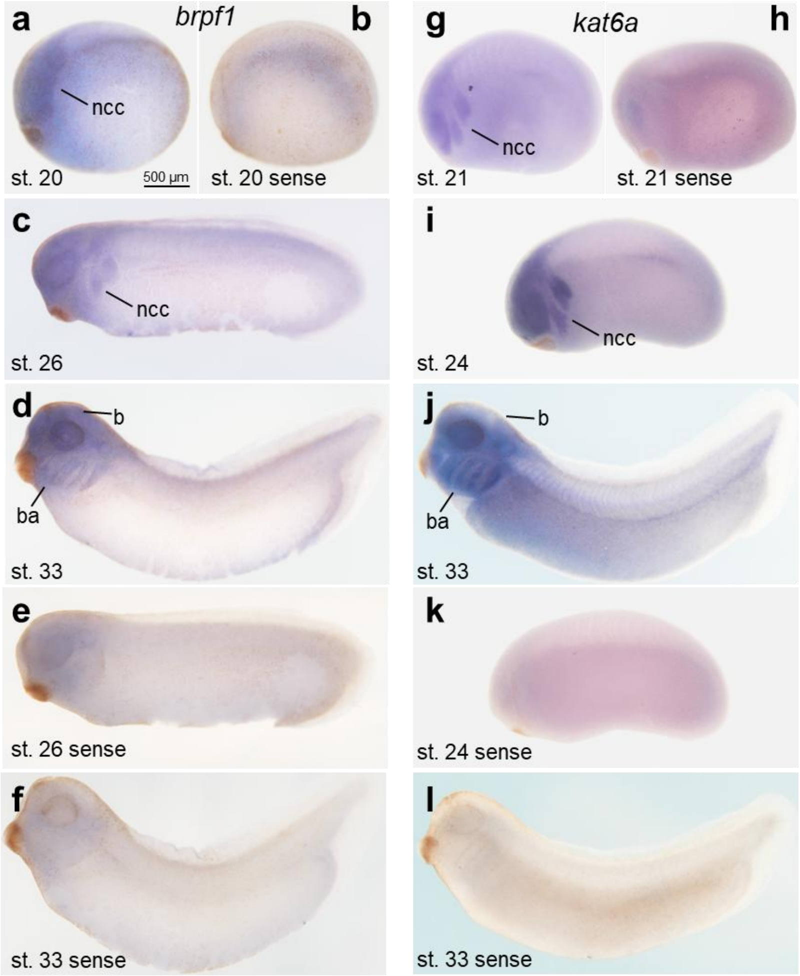

Suppl. Fig. S1 *Brpf1* and *kat6a* expression pattern in *Xenopus laevis* analyzed by whole mount *in situ* hybridization. All embryos are shown from a lateral view. **a-f** *In situ* hybridization for *brpf1*. **a** stage 20 embryo. **b** stage 20 embryo, hybridized with a *brpf1* sense control. **c** embryo at stage 26. **d** embryo at stage 33. **e,f** embryos hybridized with a *brpf1* sense control, stage 26 and 33. **g-l** *Kat6a in situ* hybridization. **g** embryo at stage 21**.** **h** stage 21 embryo, hybridized with *kat6a* sense control. **i** stage 24 embryo. **j** embryo a stage 33. **k,l** embryos hybridized with a *kat6a* sense control, stage 24 and 33. *ba* branchial arches, *b* brain, *ncc* neural crest cells
