# Supplementary material for: FBRSL1 regulates the expression of chromatin regulators BRPF1 and KAT6A
Source: Hum Genet. 2025 Jul 14;144(8):809–26. doi: 10.1007/s00439-025-02760-y (PMC12449339; doi:10.1007/s00439-025-02760-y)
Supplement: Supplementary file 5 — Supplementary material 3 (PDF 408.7 kb) [file 439_2025_2760_MOESM5_ESM.pdf]

FBRSL1 regulates the expression of chromatin regulators *KAT6A* and *BRPF1*

Human Genetics

Gina Kastens[1], Hanna Berger-Santangelo[2], Sarah Gerstner[2], Roser Ufartes[1,3], Annette Borchers[2]*, Silke Pauli[1]*

[1]Institute of Human Genetics, University Medical Center Göttingen, Heinrich-Düker-Weg 12, 37073 Göttingen, Germany

[2]Department of Biology, Molecular Embryology, Philipps-University Marburg, Karl-von-Frisch Str. 8, 35043 Marburg, Germany

[3]Synaptic Systems GmbH, Rudolf-Wissell-Straβe 28a, 37079 Göttingen, Germany.

*Corresponding authors

Silke Pauli

E-mail address: silke.pauli@med.uni-goettingen.de

Annette Borchers

E-mail address: borchers@uni-marburg.de, ORCID-ID: 0000-0002-2524-5384

Suppl. Table 4 List of the top 15 enriched motifs in FBRSL1 peak sequences identified by HOMER motif analysis.

Motif	Name	*p*-value rep #1	*p*-value rep #2
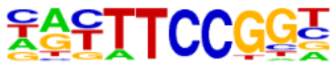	Elk4(ETS)/Hela-Elk4-ChIP-Seq(GSE31477)/Homer	1e-33	1e-14
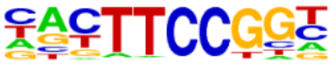	Elk1(ETS)/Hela-Elk1-ChIP-Seq(GSE31477)/Homer	1e-30	1e-11
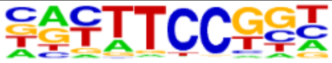	Fli1(ETS)/CD8-FLI-ChIP-Seq(GSE20898)/Homer	1e-26	1e-11
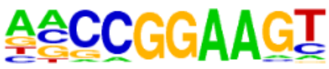	ELF1(ETS)/Jurkat-ELF1-ChIP-Seq(SRA014231)/Homer	1e-26	1e-9
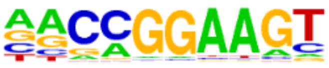	GABPA(ETS)/Jurkat-GABPa-ChIP-Seq(GSE17954)/Homer	1e-22	1e-9
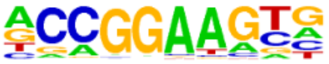	ETV4(ETS)/HepG2-ETV4-ChIP-Seq(ENCODE)/Homer	1e-21	1e-9
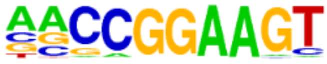	ETS(ETS)/Promoter/Homer	1e-21	1e-9
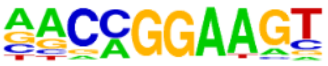	ETV1(ETS)/GIST48-ETV1-ChIP-Seq(GSE22441)/Homer	1e-18	1e-7
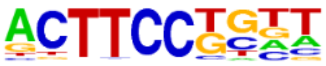	Elf4(ETS)/BMDM-Elf4-ChIP-Seq(GSE88699)/Homer	1e-11	1e-4
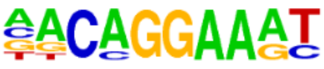	EWS:FLI1-fusion(ETS)/SK_N_MC-EWS:FLI1-ChIP-Seq(SRA014231)/Homer	1e-11	1e-5
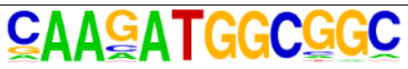	YY1(Zf)/Promoter/Homer	1e-8	1e-4
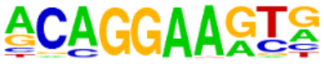	ERG(ETS)/VCaP-ERG-ChIP-Seq(GSE14097)/Homer	1e-7	1e-4
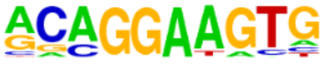	ETS1(ETS)/Jurkat-ETS1-ChIP-Seq(GSE17954)/Homer	1e-7	1e-4
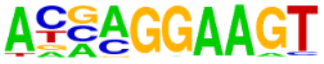	ELF5(ETS)/T47D-ELF5-ChIP-Seq(GSE30407)/Homer	1e-7	1e-3
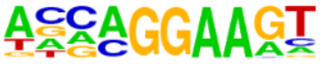	EHF(ETS)/LoVo-EHF-ChIP-Seq(GSE49402)/Homer	1e-6	1e-5
